# Appendix perforation in appendix duplication in a man: a case report

**DOI:** 10.1186/1752-1947-5-162

**Published:** 2011-04-22

**Authors:** Emel Canbay, Emel Akman

**Affiliations:** 1 General Surgery, Basaksehir State Hospital, Istanbul, 34230, Turkey; 2 Pathology, Van Yuksek Ihtisas Hospital, Van, Turkey

## Abstract

**Introduction:**

Although appendix duplication is diagnosed as a rare congenital anomaly of the alimentary tract in childhood, a few adult cases have also been reported. Here we report a case of appendix duplication with perforated appendicitis co-existing with acute appendicitis in an adult patient.

**Case presentation:**

A 33-year-old Caucasian man was admitted to our Emergency Department with right-sided lower-quadrant pain that we explored for presumed complicated appendicitis. On exploration, a perforated inflamed appendix was found coexisting with a second inflamed appendix which was subserosal and retrocecal. Appendectomies were performed, and the pathological examination confirmed the signs of acute inflammation in both appendixes.

**Conclusion:**

Surgeons in emergency services should be aware of anatomical anomalies such as duplication and malposition of the appendix, even in patients with a history of previous appendectomy, because misdiagnosis of appendix duplication may lead to a poor clinical outcome and medicolegal issues.

## Introduction

Appendix duplication is an extremely rare congenital anomaly that is seen in 0.004% to 0.009% of appendectomy specimens [1,2]. Even though the abnormality is rare, the complications that might arise from an unidentified duplicate appendix may have serious, life-threatening consequences for the patient. In patients with appendix duplication, it has been reported that acute appendicitis occurred in one [3] or both [4] appendixes and as long as six years after the first appendectomy [5]. Pre-operative diagnosis of appendix duplication is often difficult, and it is usually determined during the operation. Here we report a case of appendix duplication with appendix perforation co-existing with acute appendicitis in an adult patient.

## Case presentation

A 33-year-old Caucasian man presented with a 48-hour history of abdominal pain that started as diffuse pain and became located in the right lower quadrant. He also experienced loss of appetite, nausea and vomiting. He had undergone no previous abdominal or pelvic surgery. His physical examination revealed tenderness in the right iliac fossa, local guarding and rebound tenderness at the McBurney point, consistent with signs of complicated acute appendicitis. His body temperature was 38°C, his pulse rate was 90 beats/minute and his blood pressure was 90/50 mmHg. The urine examination result was normal. Laboratory investigations, including serum electrolyte levels and complete blood count, were within normal limits, except for a moderately elevated white cell count (14,000/mm^3^). Plain chest and abdominal radiography showed no abnormal signs.

**Figure 1:**
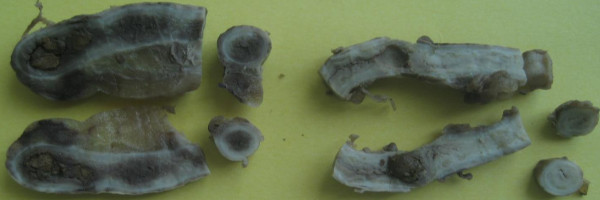
Macroscopic view of appendix duplication and obstructive fecoliths in the lumina.

Laparotomy revealed a moderate amount of purulent fluid localized in the right lower quadrant of the abdomen and perforation at the base of the appendix located in the usual place. The second appendix was dilated, inflamed and located retrocecally and subserosally. Each appendix has its own mesoappendix and its own blood supply derived from appendicular arteries, both of which were given off from the ileocolic artery. Appendixes were mobilized, both appendiceal arteries were ligated and the appendiceal stumps were managed with ligations and inversions using purse strings. A drain was placed into the rectovesical sac. The skin and subcutaneous tissues were left open and then were closed with primary sutures on post-operative day five. The macroscopic features of both appendixes are shown in Figure 1. Both appendixes were obstructed by fecoliths. Microscopic examination of both appendixes revealed prominent lymphoid follicles, necrosis and the inflammatory reactions. The patient's recovery was uneventful, and informed consent was obtained for his participation in this case study.

## Discussion

Gastrointestinal duplication is a rare congenital anomaly, and more than 80% of the patients present before the age of two years with acute abdomen or bowel obstruction [6]. Appendix duplications were first classified by Cave in 1936 [7] according to their anatomic location. This classification system was updated and modified in 1963 by Wallbridge [8]. After the most quoted version [8], two more types of appendix anomalies (Table 1) also have been described [9,10].

**Table 1:** Modified Cave-Wallbridge classification

Classification of types of appendix duplication	Features
A [7]	Single cecum with various degrees of incomplete duplication
B1 (bird type) [8]	Two appendixes symmetrically placed on either side of the ileocecal valve
B2 (tenia coli type) [8]	One appendix arises from the cecum at the usual site, and the second appendix branches from the cecum along the lines of the tenia at various distances from the first
B3 [2,3]	One appendix arises from the usual site, and the second appendix arises from the hepatic flexura
B4 [2,3]	One appendix arises from the usual site, and the second appendix arises from the splenic flexura
C [8]	Double cecum, each with an appendix
Horseshoe appendix) [9]	One appendix has two openings into a common cecum
Triple appendix [10]	One appendix arises from the cecum at the usual site, and two additional appendixes arise from the colon

In our case, a type B2 appendix anomaly (Cave-Wallbridge classification) was encountered. This duplication is reported as developing from the persistence of the transient cecal protuberance of the sixth embryonic week [6-8]. The diagnosis was evaluated according to the Alvarado Scale on the basis of clinical examination and laboratory findings [11].

Explorative laparotomy was performed in our patient. Laparotomy has also been performed in patients described in other studies [3,4]. However, Travis *et al. *[5] preferred laparoscopy for the diagnosis in their patient who had undergone a previous appendectomy. Diagnostic laparoscopy as a minimally invasive technique is now the most widely used and preferred technique compared with laparotomy. Advanced radiologic techniques can be useful for the diagnosis of intra-abdominal pathology before surgery. Even though computed tomography scans are not useful [5] and are not used in all cases [3], the diagnosis of appendix duplication with inflammation can be made [4]. Misdiagnosis and mismanagement are common occurrences in such cases because of the rarity of the appendix anomalies. As in our case, previously reported appendix duplications have also been diagnosed during surgery in these patients [2]. It has been reported that the second appendix could be histologically normal during the appendectomy [3,12,13], which leads to a delay in misdiagnosis. Delays in diagnosis of a second appendix may lead to increased risk of perforation [5]. Duplication of the appendix should be considered in all cases of lower abdominal pain, even if the patient reports a previous appendectomy. An inflammatory mass associated with a solitary cecal diverticulum may have a similar clinical presentation and may be discovered together with appendix duplication during a laparotomy [8]. However, it may not be possible to differentiate them clinically, and the distinction may be made only by histologic examination of the specimen. The wall of a cecal diverticulum lacks lymphoid tissue that is typically present in the vermiform appendix specimen [8]. Appendix duplication may also present as a constricting lesion of the ascending colon and mimic a colonic adenocarcinoma [14].

## Conclusion

Surgeons who deal with cases including a previous appendectomy in emergency services should be aware of the anatomic anomalies such as appendix duplication and malposition of the appendix, because misdiagnosis of appendix duplication may lead to a poor clinical outcome.

## Consent

Written informed consent was obtained from the patient for publication of this case report and accompanying images. A copy of the written consent is available for review by the Editor-in-Chief of this journal.

## Competing interests

The authors declare that they have no competing interests.

## Authors' contributions

EC was the surgeon who performed the operation and close follow-up of the patient and was the major contributor to writing the manuscript. EA carried out the histopathologic evaluation of specimens and interpreted the patient samples. Both authors read and approved the final manuscript.
